# IoTMapper: A Metrics Aggregation System Architecture in Support of Smart City Solutions

**DOI:** 10.3390/s22197484

**Published:** 2022-10-02

**Authors:** João Pedro Vitorino, Nuno Cruz

**Affiliations:** 1 Future Internet Technologies—FIT, Instituto Superior de Engenharia de Lisboa—ISEL, Instituto Politécnico de Lisboa, 1500-335 Lisbon, Portugal; 2 LASIGE, Faculdade de Ciências, Universidade de Lisboa, 1749-016 Lisboa, Portugal

**Keywords:** smart cities, internet of things, FIWARE, Apache Kafka, LoRaWAN, low-power wide area network (LPWAN)

## Abstract

Smart cities are, nowadays, an unavoidable and growing reality, supported on software platforms that support city management, through the processing and presentation of a large number of data, obtained from sensors used throughout the cities. Low-power wide area networks (LPWAN) leverage the sensorization process; however, urban landscape, in turn, induces a high probability of change in the propagation conditions of the LPWAN network, thus requiring active monitoring solutions for assessing the city LPWAN network condition. Currently existing solutions usually consider the existence of only one type of LPWAN network to be monitored. In this paper, an architecture for aggregation of metrics from heterogeneous LPWAN networks is presented. The architecture, named IoTMapper, combines purpose build components with existing components from the FIWARE and Apache Kafka ecosystems. Implementation details for the LPWAN networks are abstracted by adapters so that new networks may be easily added. The validation was carried out using real data collected for long-range wide-area network (LoRaWAN) in Lisbon, and a simulated data set extrapolated from the collected data. The results indicate that the presented architecture is a viable solution for metrics aggregation that may be expanded to support multiple networks. However, some of the considered FIWARE components present performance bottlenecks that may hinder the scaling of the architecture while processing new message arrivals.

## 1. Introduction

According to the United Nations, more than half of the world’s population resides in urban areas, with an upward trend [1]. This fact, coupled with the overall growth of population in the world, results in significant development on the size and complexity of cities, which presents a significant challenge for the management methods used in cities [2]. New strategies are required for information gathering and decision making in the efficient management of available resources, promoting an increase in the number of cities that implement so-called smart city solutions, which in turn support different verticals of municipalities’ intervention.

The term smart city is understood as the sensorization, in the context of the internet of things (IoT), of urban areas in order to, through analysis of the data collected, improve the living conditions of citizens as well as the quality and sustainability of services provided.

The sensorization of city infrastructure results in the collection of significant volumes of data, possibly critical in the new management schemes defined for the smart city, that needs to be transmitted in a timely, reliable, and cost-effective fashion. As such, the sensors used in data collection require means for periodic and consistent communications. This communication requirement is addressed by low-power wide-area networks (LPWAN).

An LPWAN is a wireless communication network optimized for long-range communication, low power consumption and low cost of the associated network interface. In order to support the smart city verticals, different LPWAN technologies have been developed. Examples of these communication protocols, in common use to support smart cities solutions [3,4,5,6], are:

- Long range wide-area network (LoRaWAN) [7,8,9]: The complementary network layer to the LoRa technology, which, by itself, only specifies the physical layer of the communications stack.
- Sigfox [6]: An alternative network with the same aim, based on a closed business model, in which the network is always supported by an operator, also called Sigfox. This is the key difference from other technologies; there is only one operator.
- Narrowband internet of things (NB-IoT) [10,11]: A technology supported by public mobile communications operators, resulting from the evolution of the widely deployed LTE (long-term evolution) technology. NB-IoT is a more recent IoT technology, its operating model is based on the conventional models of public mobile operators, and is based on a subscription.

LPWAN technologies, in particular LoRaWAN, will be further described in Section 2.1.

The validation of theLoRaWAN architecture present in Lisbon is particularly considered. This particular deployment is freely available, and there is a previous body of work in the study and development of solutions using this deployment, such as in urban waste management [3]. The authors, however, believe that their approach is applicable to any urban environment. Additionally, solutions using LoRaWAN have already been noted to attract particular interest among researchers and in the deployment of new smart cities solutions [8].

In the context of LPWAN networks in an urban environment, whose goal is to support smart cities solutions, the authors consider a number of specific challenges that require regular assessment of network coverage:

1. The urban landscape may change, for example, the construction of new buildings, which affect the propagation characteristics of existing networks.
2. The continuous expansion of the city, both in terms of population and occupied geographical area, may increase the pressure such that networks are subjected through a higher traffic volume and device number, which was an issue identified in previous research such as in [2].

While not specific to the context of smart cities, the authors also specifically consider that, in the same area, there may coexist multiple LPWAN networks, each requiring adequate monitoring. Thus, the need arises for a solution that can assess the quality and coverage offered by multiple existing networks in a continuous fashion in order to identify potential gaps in geographic coverage, or, areas with heavy saturation, leading the effort of maintenance and future investment in the adaptation and expansion of existing networks.

The presented architecture makes specific usage of components included in the FIWARE catalog [12]. The FIWARE project was developed under the funding of the European Commission to create a catalog of reusable *open source* components that can be assembled together, and with other third-party components, to build IoT platforms that support the development of smart solutions in a standardized, faster, easier and cheaper manner. As part of the stated objective, a number of components for integration of external data sources and processing components were developed. Their usage was considered on the design of architecture. Additionally, the usage of data models conforming to the NGSI-v2 application programming interface (API) [13] was considered, enabling the reuse and integration of metrics data in a standardized fashion. The FIWARE project is further described in Section 2.2.

Two existing solutions, TTNMapper [14] and HeliumMapper [15], were identified. However, both solutions only allow for monitoring LoRaWAN deployments, and neither make use of the FIWARE project. They are further presented in Section 2.3.

### 1.1. Contributions

The major contribution in this paper is the proposal, implementation and validation of the architecture for a new *open source* based system capable of receiving, aggregating and presenting metrics on the coverage quality of LPWAN networks. This system is named IoTMapper. The proposed architecture presents an end-to-end scalable solution, from reception of metrics from specified data sources to their availability to end users and external system, through a web-based application programming interface (API), while remaining generic enough such that support for additional LPWAN networks can be added with minimal development effort by adding new receivers and messages parsers. A reference implementation and installation scripts that may be used in deployment and further development of monitoring solutions based on IoTMapper were developed. Furthermore, the validation of the implementation as well as the qualitative and quantitative evaluation of the overall system performance is presented.

Of particular consideration is the evaluation of components from the FIWARE [12] catalog when put under different load volumes, which have not been previously considered in previous research. Additional contributions relate to the proposal of new FIWARE data models to represent the aggregated metrics and associated contextual information in a standardized and reusable manner.

The objective of this is not to advance the state of the art on coverage models for LPWAN networks, but to apply and validate existing technological components in the development of a new integrated solution. Additionally, it is noted that the work does not include the development of physical IoT devices used in metrics collection, but is focused on the infrastructure that supports such devices.

### 1.2. Organization of the Document

The rest of this document is organized as follows: in Section 2, additional background information and related work are discussed; in Section 3, the proposed architecture and reference implementation details are presented; and the obtained results are presented and discussed in Section 4. Concluding remarks and future work directions are given in Section 5.

## 2. Background and Related Work

### 2.1. LPWAN for IoT and Smart Cities

In the construction of smart cities, one of the main objectives is making city services more flexible and reactive. This objective motivates the integration of numerous vertical applications into the existing infrastructure of the urban areas. A vertical refers to a complete IoT solution, from the data collecting devices and actuators to the communication layer and processing solutions. As already noted in the introduction, the need for this kind of integration is seeing an upwards trend, being associated with significant population growth in urban areas [1,2].

The IoT devices used, due to their nature, have limited computational resources and battery lives that they need to conserve. While individual measures typically are only a few bytes in size, the total volume of information that is produced by these devices may be too significant to be processed locally with the limited resources available. It may also need to be jointly analyzed to produce any meaningful result, or need to produce alerts in a timely manner. As such, in support of smart cities verticals, a quality communication layer is need that allows the efficient transmission of produced data.

The term low-power wide-area networks (LPWAN) refers to a set of wireless communication protocols mainly characterized for offering a combination of high range, low energy consumption, and low manufacturing costs for the associated devices as seen in [5,16,17]. This set of characteristics make the usage of LPWAN a natural fit for IoT applications, including integration scenarios for smart cities solutions. In fact, there is a wide body of work on the applicability of LPWAN technologies in smart cities scenarios. In [8], the authors conducted a survey on the published scientific articles on LoRaWAN-based solutions used in smart cities. They identified a large variety of verticals where LoRaWAN is in use, from energy management to waste management, and predicted a growth of 10% per year until 2025 in new LoRaWAN solutions. LPWAN deployments follow a star network topology, where messages sent by devices are received by networking equipment, typically designated as gateways.

Some LPWANs, such as NB-IoT, depend on a licensed spectrum in its operation, particularly bands already reserved for mobile communications. As such, its availability depends on Mobile Communications Operators (MCO) that reuse existing cellular base stations. For these LPWANs, the metrics that can be collected without the collaboration of the corresponding MCO are limited to those observable by the equipment responsible for sending the measurement messages, as seen in [18,19].

#### LoRaWAN

**Figure 1:**
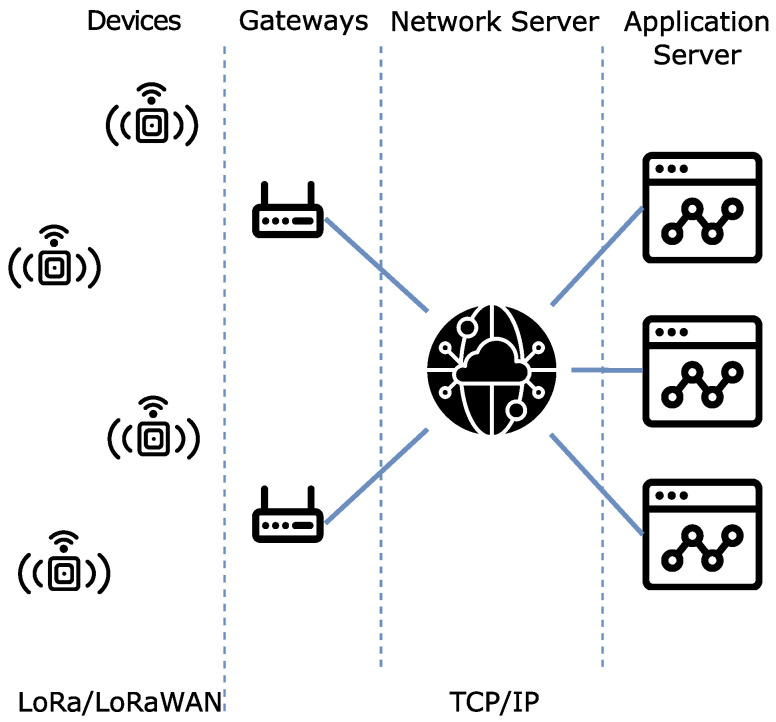
High-level view of the LoRaWAN architecture.

In LoRaWAN deployments, the underlying physical protocol layers, LoRa, is defined over an unlicensed radio spectrum and is available for general use, both for public, open networks, and for private networks on the sale of commercial solutions. LoRa, as standardized by the LoRa Alliance [7], uses frequencies around 868 MHz (for Europe), 915 MHz (for North America and Brazil) and 433 MHz in multiple countries. In LoRaWAN architectures the gateways have a connection to a network server that handles reception control and other network issues before passing them to an application server that handles routing of messages. In this model (visible in Figure 1), LoRaWAN accepts that the same messages may be received by multiple gateways. Since all receptions are valid, the network sever merges them into a single message, with metadata for all deliveries. There are multiple *open source* implementations of LoRaWAN available. The things network (TTN) offers a community developed and operated solution for a LoRaWAN server as a service that gateways and devices operators may freely use [3,20,21,22].

The open specification used by LoRaWAN allows for access to meaningful metrics on network coverage, if reported by the gateway, such as received signal strength indication (RSSI), signal-to-noise ratio (SNR), frequency/channel used, and possibly the location of the gateway.

### 2.2. The FIWARE Framework

FIWARE [12] is a project, under funding from the European Commission for the development of an IoT framework offering a catalog of reusable *open source* components that can be assembled together and with other third-party components to build IoT platforms that support the development of smart solutions in a faster, easier and cheaper manner.

For the stated purpose, FIWARE defines a set of generic enablers (GE), software components with well-defined responsibilities that offer their capabilities as services. These GEs can be combined to compose functioning solutions. To permit interoperability between GEs, they all follow a common context management model, under the NGSI-v2 REST application programming interface (API) [13], or, the NGSI-LD API. These APIs include the definition of a common and reusable, but expandable, set of data models,

The primary enabling component offered by FIWARE is the Orion Context Broker (OCB) that offers a publish/subscribe service for persistence of the current context, as a set of entities. This managed context can be easily shared between GEs and external components, allowing for simple interoperability. The subscription aspect of OCB is assured by an asynchronous “notification” system. The notifications are triggered by changes in the state of entities, resulting in a hypertext transfer protocol (HTTP), or MQ telemetry transport (MQTT) message to another component. The major limitation of OCB is that only the most current version is persisted in its backing store. This limitation can be overcome by using an additional GE with historical data capabilities, such as STH Comet, or QuantumLeap.

The IoT Agents GEs are a set of related GEs, using a common base library, to offer support for different IoT communication protocols. The IoT Agents map receives messages from NGSI entities and inserts them into the OCB. The IoTAgent-LoRaWAN that offers integration for LoRaWAN, including specific support for TTN application server, was selected as the implementation for validation.

The FIWARE catalog also offers GEs for integration with external systems. Cygnus is an example of such a GE that can be used to export data both to external storage (PostgreSQL, MySQL, MongoDB or AWS DynamoDB), or asynchronous publish/subscribe (Apache Kafka) that can be integrated with further processing frameworks.

### 2.3. Related Work

There is an extensive and growing body of work on LPWAN-based solutions for smart cities and other areas, such as smart agriculture, highlighting their importance in the literature. Considering just LoRaWAn solutions, there were 30 published articles in 2016, increasing to about 400 in 2018 [8]. While the body of work is extensive, the vast majority of identified published articles focus either on comparing LPWAN technologies, on the design of solutions using LPWAN technologies, or on assessments on the quality of LPWAN that simply make a one-off analysis of the deployment. There is limited research focusing on solutions to analyze the quality of the LPWAN deployment themselves in a continuous manner.

Regarding work that analyzes the quality of LPWAN deployments, in [23], the authors performed a theoretical study of a LoRaWAN network for the simulated model of a typical urban environment. In [9], the authors reported on a real use case of a LoRaWAN network installed in the city of Southampton in the United Kingdom, by analyzing collected data at a later date. For [24], the authors performed a long-term measurement campaign for NB-IoT, concluding that the reference signal receive power (RSRP) varies even for stationary targets. They then presented a doubly stochastic Markov chain model, from the time-dependent statistical characteristics, demonstrating that such an approach may be used for the optimization of modern LPWAN technologies, given a set of collected data on the deployments. A coverage assessment method was presented in [25]. This assessment starts with a large-scale measurement campaign for NB-IoT, Sigfox, and LoRaWAN. Using the collected data, the authors proposed a procedure for identifying the minimal set of points for evaluating the coverage offered by an LPWAN network, for which there is no information on base stations. Ref. [26] presented another measurement campaign, for Nb-IoT, and made the collected data set available, while identifying the impact of different deployment decisions.

None of the referenced research articles proposed a system architecture that actively supports their data collection process.

Regarding work that offers more active monitoring, in [18], the authors presented NBPilot, an embedded system for local quality analysis in NB-IoT networks, based on the extraction and processing of network signaling messages, but without the definition of a support infrastructure for the collection and aggregation of metrics. Ref. [19] described NBViewer, a demonstrator for a software tool set that locally collects and analyzes data for a single NB-IoT device.

None of the presented solutions proposed an architecture for collecting data from multiple devices at scale.

Considering existing *open source* solutions already available for use, two can be identified: TTNMapper [14] and HeliumMapper [15]. Other commercial solutions are also available, but due to their *closed source* nature, they could not be appropriately considered [27].

TTN Mapper is a LoRaWAN mapping solution, originally in versions 1 and 2, specific to the TTN community application server [20]. In version 3, support for other LoRaWAN servers was added, namely ChirpStack and Helium. TTN Mapper offers a microservices-oriented architecture with communication between components guaranteed through the use of advanced message queuing protocol (AMQP) asynchronous message queues, implemented with RabbitMQ. Reception of the metrics is performed over HTTP through integration with the TTN server, which forwards data from devices that participate voluntarily. The components sequentially perform data processing, aggregation, and persistence steps in a PostgresSQL database. The performed aggregation joins the measurements into geographic cells. The implemented dashboard provides visualization of the current state of the network as a heat map. The HeliumMapper project also presents a platform for visualizing the quality associated with a specific LoRaWAN application server. Similar to TTNMapper, metrics are received through HTTP by integrating with Helium applications that provide reports about their devices voluntarily. The reception of reports is followed by aggregation of the metrics into H3 geographic cells, originally developed at Uber [28], persisted in a PostgresSQL database. The H3 cells are a hierarchical index that groups coordinates into a continuous grid of hexagonal-shaped area blocks with a unique identity. The system collects, as metrics, information about received signal strength indication (RSSI), signal-to-noise ratio (SNR), number of *gateways* and distance from the equipment to the gateways. The dashboard is implemented as a heatmap, divided by H3 cells.

The solutions already identified (TTNMapper and HeliumMapper) allow to answer some of the identified challenges, but they fail to answer all of them. In particular, they focus on supporting a single LPWAN network, in both cases a set of specific application servers for LoRaWAN. Additionally, neither solution uses any component related to FIWARE.

To the best of the authors’ knowledge, there is no previous work that uses FIWARE components to gather metrics on the LPWAN deployments themselves, in opposition to metrics about the IoT devices or the monitored environment. There is, as well, no *open source* system available that processes metrics on multiple LPWAN networks in a close to real time manner. In this context, the authors consider that there exists the need to design and validate a new *open source* solution that considers the existence of multiple LPWAN networks as a starting point, and evaluates the feasibility of using existing FIWARE components for context management and integration with external data sources.

## 3. Materials and Methods

### 3.1. Methodology

Following from the presented background and related work, the developed work was guided under the following phases: problem identification, listing of requirements, design of system architecture, implementation considerations, integration of components, and validation of the designed system.

### 3.2. Problem

As presented in Section 1 and Section 2, the problem to address revolves around the growing need to support smart cities solutions in a environment that may change over the life of the deployment, and include multiple low-power wide area networks (LPWAN). In this scenario, the main objective is to develop a scalable solution that may actively monitor the state of LPWAN networks. Additionally, it is a specific objective that the proposed architecture makes use of FIWARE components.

### 3.3. Requirements

Considering the objectives presented above, the requirements of the system are as follows:

1. The system must make use of components from the FIWARE catalog;
2. The system must be scalable, in such a way that it may be scaled to support a wide geographic area, with high volumes of messages and multiple LPWAN networks:
  (a) The system must at least implement support for long-range wide-area network (LoRaWAN) over the things network (TTN);
  (b) The design choices must not limit future support for additional LPWAN networks, deployed using distinct protocols.
3. The system must join metrics by some geographic area and aggregate them, obtaining the average value for each collected metric;
4. The system should not lose more than 1% of messages received from data sources:
  (a) It is acceptable that this introduces significant latency in updating aggregated metrics, in the order of a few seconds.
5. The system must make the aggregated metrics available, both to external systems through a web application programming interface (API), and to the final user through a graphical interface.

### 3.4. Design of the System Architecture

The proposed architecture has as an obligatory requirement that it must use components from the FIWARE catalog for system integration and context management. Naturally, such an architecture is built around the usage of the Orion Context Broker (OCB).

**Figure 2:**
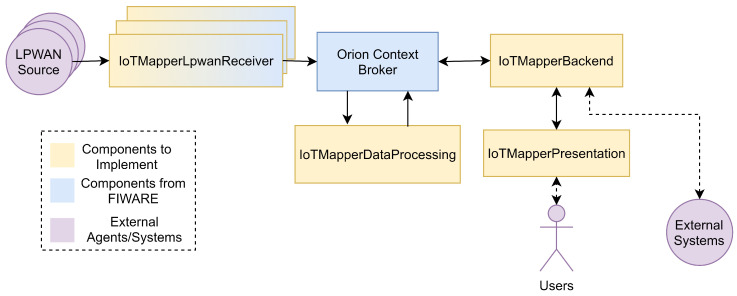
High-level view of system architecture.

Consider Figure 2 that presents a high-level view of the proposed architecture for IoTMapper. The functional blocks of the system can be summarized as follows:

- The low-power wide-area networks (LPWAN) sources represent any external system, or internet-of-things (IoT) device that reports metrics about an LPWAN network.
- The IoTMapperLpwanReceivers are the set of components suitable for one or more LPWAN networks, mediating the reception and insertion of metrics. It may be any component capable of using the NGSI-v2 API, but the FIWARE catalog already offers some adequate components, namely the IoTAgents.
- The Orion Context Broker (OCB) performs the role of manager of the context data, composed of initial metrics events and the obtained aggregations, allowing the integration of other FIWARE components according to the NGSI-v2 API.
- The IoTMapperDataProcessing obtains the initial metrics from the OCB, processing them in near real time as a stream of events to be processed, filtered and aggregated into OCB as the final metrics entities.
- The IoTMapperBackend intermediates access to the managed context, allowing both external systems and the IoTMapperPresentation to consume the aggregated metrics entities, while abstracting implementation details, such as the FIWARE services used for the context separation of entities.
- The IoTMapperPresentation permits an initial and simplified overview of the aggregated metrics as a heatmap presenting the relative quality of the collected metrics.

### 3.5. Implementation Considerations

Considering the high-level view presented in Section 3.4, a reference implementation was developed as validation of the proposed architecture.

All components, either newly developed or already existing, were containerized with the specific intent of orchestrating the deployment of the entire system using Kubernetes [29,30,31]. The installation and initialization process was unified under a single Ansible [32] playbook, allowing the deployment of the entire system with a single command.

#### 3.5.1. IoTMapperLpwanReceivers

These receivers are the input point for all received data; as previously stated, they may be any component capable of using NGSI-v2 to insert metric reports into the OCB. For validation of the purposed architecture, the set of IoTAgent components offered by the FIWARE catalog was chosen. In particular, for the development of the reference implementation and support of LoraWAN networks, the IoTAgent-LoRaWAN component was chosen. The IoTAgent-LoRaWAN implementation was modified to add a new mapping mode, allowing for the consumption of the entire LoRaWAN message, including headers that contain the required metrics, in opposition to existing modes that consume only the payload sent by the IoT devices serviced by the LoRaWAN network.

At this point, the need was identified for a common format that may be used to represent the metrics reports inserted into the OCB, irrespective of their source. The choice was made to keep details regarding the processing of metrics, specific to each LPWAN network, maximally contained to the IoTMapperDataProcessing component, representing a single point to be altered and limiting further alterations to FIWARE components. To answer this question, a new data model, the *MetricsReport*, was defined as detailed in Appendix A.1.

#### 3.5.2. Orion Context Broker—Layers for Separation

When considering the definition of data flows in the system of the proposed architecture, two flows can be identified:

1. A input flow: from the reception of metrics reports to the creation and update of the metrics aggregations;
2. A exit flow: that allows the consumption of aggregations through IoTMapperBackend and IoTMapperPresentation.

Both flows have the Orion Context Broker as the common connecting point. They, however, represent two significantly distinct use cases. The input flow is a more write-heavy scenario, with reports arriving and updates to aggregations additionally making use of NGSI-v2 asynchronous notifications. The exit flow is almost exclusively composed of reads of the aggregated metrics from the managed context.

Considering the two different usage flows under the same architecture, for implementation purposes, the separation of the system in two distinct *layers*, each with its own instance of the OCB, was considered, resulting in an *input layer* that has the IoTMapperLpwanReceivers and IoTMapperDataProcessing and an *exit layer* with IoTMapperBackend and IoTMapperPresentation.

#### 3.5.3. IoTMapperDataProcessing

Similar to the case for IoTMapperLpwanReceivers, a new data model is required to represent the results of metrics processing and aggregation, as outputs of IoTMapperDataProcessing to be inserted into OCB. The defined model, a *MetricsAggregation*, is described in Appendix A.2.

While a simpler approach may have been the creation of a single service that processes the metrics and emits the resulting *MetricsAggregations* backed by a storage solution, this option was discarded in favor of a more complex approach, using a combination of an Apache Kafka [33,34,35] and Kafka Streams [36] applications, with Cygnus from the FIWARE framework, writing *MetricsReports* to topics in Apache Kafka.

This choice is motivated by a number of factors. Firstly, the objective of creating a processing system with low loss of messages, capable of handling a variable volume of messages and that can be easily extended with additional functionality (new parsers for new LPWAN networks). Apache Kafka offers *exactly once* semantics, which can be advantaged by Kafka Streams applications. Each such application is a Java application, using the provided library to define streams of processing steps to be applied to messages, while offering such characteristics as progress check pointing, load balancing and the grouping of messages according to a key.

Secondly, the usage of an asynchronous publish/subscribe framework naturally extends the asynchronous publish/subscribe behavior of the notification mechanism offered by OCB, with Apache Kafka being the only such framework supported by a FIWARE component (Cygnus).

Lastly is the possibility of using the Apache Kafka brokers as an aggregation point for data originating from multiple OCB instances in a scaling environment.

This results in the division of the IoTMapperDataProcessing component in a number of interrelated components:

- A FIWARE Cygnus component that processes notifications received from the OCB, inserting them into a topic in the Apache Kafka broker.
- The Apache Kafka brokers that handle the distribution of messages between components, while ensuring *exactly once* message delivery guaranties.
- The actual implementation of the IoTMapperDataProcessing logic, a set of Kafka Streams components, responsible for all processing of messages and the calculation of aggregated metrics. The processing is divided into steps that are check pointed into Apache Kafka topics.
- A Kafka Connect [36,37] component, offering a newly implemented connector capable of using NGSI-v2, notably including the batching of entities updates. As the *MetricsAggregations* are added to each output topic, they are inserted into the OCB.

The enumerated components, in the presented order, also serve as a summary of the previously referred input flow, lacking only a reference to the initial reception of messages by the IoTMapperLpwanReceivers.

The Kafka Streams applications that implement the logic of the IoTMapperDataProcessing were designed so that, while possessing a common core of reusable logic, a new specialization of the component, supporting a new LPWAN network, could be added as required. Additionally, under this architecture, it would be reasonable to developed new steps to perform additional analysis of the streams and aggregated metrics.

**Figure 3:**
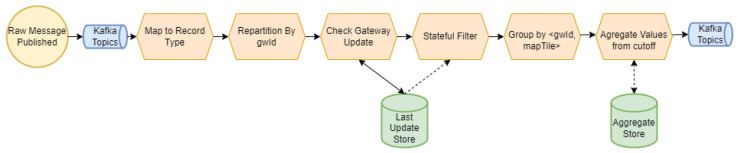
Sequence of steps performed by IoTMapperDataProcessing components.

The common logic defined for IoTMapperDataProcessing components is presented in Figure 3.

The sequential logic can be divided into steps such that the following hold:

1. Mapping: Reading and mapping from a generic format, a *MetricsReports*, to a type specific to each LPWAN, a *IMetricsRecord*.
2. Repartition: Initially, the messages do not have an associated key. The correct distribution of messages is assured by extracting from each message a gateway identifier (GwId) to be used as a key.
3. Gateway update: The collected metrics are directly correlated with the test conditions (environment, weather, hardware, etc.). Any change on these will probably be reflected on the extracted samples, and one would need to redo the network surveys so that they are only valid while channel characteristics are reasonably unaltered. IoTMapperDataProcessing checks, for each new *IMetricsRecord*, a set of mechanisms to verify their validity. As a default, a gateway may not move more than 100 meters from the first observed location, and it is also possible to configure a time to live for *IMetricsRecord*. Whenever channel characteristics are deemed to have changed, a new aggregation is started. Another parameter of interest could be the time since the start of the current aggregation.
4. Filtering: The message flow is filtered in order to exclude out-of-order messages posterior to the last change.
5. Metrics grouping: The metrics are grouped in geographic areas (designated *mapTiles*), obtaining pairs <gwId,mapTile>. The mapTiles are formed as GeoHashes [38], due to the wide availability of compatible libraries.
6. Metrics aggregation: The obtained set of metrics, already filtered, is aggregated obtaining the final internal representation, a *IMetricsAggregate*. The logic to aggregate metrics is specific to each LPWAN network.
7. Writing the results: The aggregations are persisted in a topic to be consumed by Kafka Connect, already formed as NGSI-v2 entities (*MetricsAggregations*).

#### 3.5.4. IoTMapperBackend

All the collected *MetricsAggregations* are collected with the objective of supporting decision making in the maintenance of LPWAN networks by presenting coverage maps for existing networks.

The IoTMapperBackend covers this objective by abstracting all implementation details that would make consumption more complex, and limiting access to non-essential details. For example, the implementation separates the context of *MetricsReports* and *MetricsAggregations* entities from different LPWAN networks by using distinct FIWARE services, a HTTP header in NGSI-v2 with mostly internal meaning. In the IoTMapperBackend, the implemented API offers access to lists of these entities through a common interface for all LPWANs that may be consumed by external systems.

The implementation was developed using Kotlin for the Spring Framework [39,40].

#### 3.5.5. IoTMapperPresentation

While the IoTMapperBackend offers access to detailed information on coverage, IoTMapperPresentation offers an initial view of the collected data. As validation of the architecture, and considering the need for a common interface for all metrics, the IoTMapperPresentation component implements a heatmap displaying the relative qualitative coverage.

The implementation was developed using Typescript for the React framework [41]. The implemented interface is shown in Section 4.1.

### 3.6. Integration for Validation

For validation of the developed architecture, the system was integrated in a laboratory environment, made available by ISEL that used three virtual machines for deployment as described in Table 1.

Latency between the machines was measured to be negligible, always inferior to 1 ms, in a simple experiment of using the ping utility between the machines.

The 1.24 version of Microk8s [42,43] was used to create a Kubernetes cluster between TFM-00 and TFM-01, while separating deployed components according to their *layer*.

### 3.7. Validation and Evaluation

The proposed system was validated by considering, in a first stage, a data set obtained from a real environment. This first data set is composed of survey messages, sent from a moving car through the city of Lisbon, Portugal, for a total of 2100 distinct messages, each being received by 2–3 gateways on average, and resulting in 3185 distinct aggregations. Each message contained the current location of the survey device in its payload. The data set was collected through the things network and stored in its raw form. This data set was collected on 4 November 2021, over a period of 4 h.

**Figure 4:**
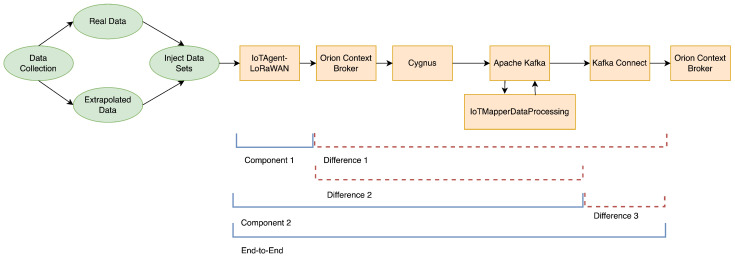
Graphical representation of the methodology framework, used during evaluation.

In a second stage, a simulated data set was generated, using the messages collected in the first stage as a base in a semi-randomized fashion. This semi-randomized component refers to the location field present in each message, which was altered in such a way that each message corresponds to a single Geohash identifier (a MapTile, presented in Section 3.5.3) for the same gateway identifier. This choice allows a direct correspondence between each message injected in the system, and the entities created in the context management solution (the OCB). Such a characteristic is important in the approach used for identifying both latency and error rates. Figure 4 presents the methodology framework used for this stage.

In order to simulate the process of collecting the messages from a real source, with controllable rates, a MQ telemetry transport (MQTT) broker was added to TFM-01 only for the second stage. Eclipse Mosquitto [44] was the chosen implementation. An auxiliary software component was purpose built to inject the second data set into the MQTT broker, according to the required rate, while inserting the current timestamp into each message. As the data set is injected into the system, the evaluation does not consider, in the current development phase, any latency that would result from each LPWAN deployment, before the messages reach IoTMapper.

Individual components were validated as development progressed. The integration of all components, including user interface, was validated for a real use case and environment during the first stage. Performance of the integrated system was collected during the second stage for a laboratory setting.

For the second stage, two simple metrics of system performance were collected: total error rate (TER) and average latency (AL).

The TER indicates the percentage of messages either dropped or that fail to be fully processed in a reasonable time period. The definition is given by

(1) $$TER=\frac{S_{i}}{n}\times 100$$

where $S_{i}$ is the count messages successfully processed in *i* seconds, and *n* is the number of messages sent to the system.

AL indicates the average delay, in milliseconds, for the group of messages successfully processed to reach the indicated component. The method used to measure end-to-end latency is dependent on the comparison of timestamps injected at different points in the system. The last timestamp, for end-to-end latency, is inserted into each *MetricsAggregation* entity by the OCB instance. Two additional timestamps are also obtained for other points. After completing each test, the timestamps were collected and compared in order to obtain the latency for each message as it passes through the system. The definition used in the comparison is given by

(2) $$AL=\frac{\sum_{t=1}^{n}|[f_{t}-i_{t}]|}{n}$$

where *n* is, again, the number of messages sent to the system. $i_{t}$ is the first timestamp, inserted into each message as it is injected into the system. $f_{t}$ is the timestamp inserted for comparison, as defined in Table 2 and Figure 4.

The measurement points were chosen with the primary objective of testing the performance of the IoTAgent-LoRaWAN, used as the IoTMapperLpwanReceiver implementation when integrating LoRaWAN networks, and as a result of the availability of the required data in each component. As there are only three possible measurement points, the set of obtained metrics was bolstered by calculating the average differences between measurement points, allowing the identification of time spent in a subset of components.

The results are presented in Section 4.

## 4. Results and Discussion

As previously stated in Section 3.7, the system was tested in two stages. Firstly, the system was validated under the environment of the city of Lisbon by accessing the coverage offered by a LoRaWAN network mounted by city authorities [2]. In the second stage, a simulated data set was used to evaluate system performance when under a controlled load. For the second stage, the fact that each message in the data set maps to a single *MapTile* and, by extension, a single *MetricsAggregation* entity for consumption, is an important characteristic that could not easily be guaranteed in a real environment. In particular, it allows for simple verification of message loss during testing, as under regular circumstances, a LoRaWAN message may be received by multiple gateways, resulting in each message being split into multiple *MetricsAggregation* entities.

### 4.1. First Stage—Validation

**Figure 5:**
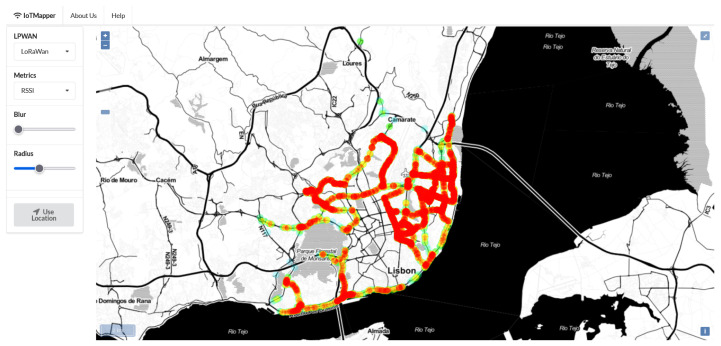
User interface developed for the IoTMapper system. Displaying relative RSSI levels for LoRaWAN in Lisbon.

Consider Figure 5 which presents the graphical user interface centered on the downtown area of Lisbon. The graphical interface presents the user with a heatmap showing relative quality for the *MapTiles* for which coverage was recorded. The left sidebar allows the user to select the displayed data set from the drop-down selections. The *LPWAN* drop-down loads the monitored low-power wide area networks (LPWAN), while the *Metrics* drop-down renders the map for the selected aggregated metric. For Figure 5, the selected combination is the received signal strength indicator (RSSI) for LoRaWAN.

This first stage allowed for the validation of the correct functioning of the developed system under a real environment.

### 4.2. Second Stage—Evaluation

Using the second data set and performance metrics as described in Section 3.7, the system was strained under different messages rates. The obtained results are as follows.

#### 4.2.1. Latency

**Figure 6:**
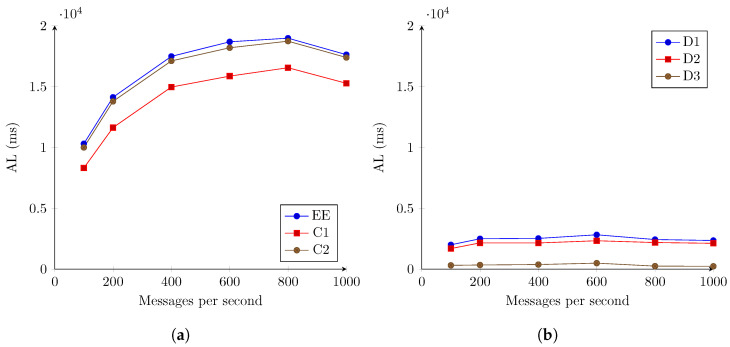
AL registered for the second stage evaluation. LoRaWAN for 1 simulated device. (**a**) Registered latencies for monitored components and end to end. (**b**) Differences in recorded latencies between components.

Figure 6 shows the variation of the average latency (AL), as defined in Equation (2), for the three measuring points and average differences between them, as defined in Table 2.

Comparing the recorded values, it is clear that the majority of the latency in the system is introduced by the interaction between the first component of the overall system, the IoTAgent-LoRaWAN, and the OCB, corresponding to the C1 measurement point. C2 and EE, which correspond to measuring points down the processing pipeline, show only minor increases in latency. It is also clear that the latency increases significantly as the number of messages per second increases. This pattern is slightly contradicted for 1000 messages per second.

While the requirements listed for the project allowed for sacrifices in the delay to make updated *MetricsAggregation* entities available for consumption, in favor of limiting loss of messages, the growing delay introduced by the IoTAgent-LoRaWAN component is not considered to be falling under the concessions made for this requirement, and presents a significant bottleneck to the usability and scalability of the system.

As the IoTAgent-LoRaWAN does not offer or use any batching mechanism, each message received results in a new, individual write into the underlying entity stored in the OCB. In the first iteration of the test (Figure 6), all the messages received belong to the same simulated device, being mapped by the IoTAgent-LoRaWAN component to the same entity.

**Figure 7:**
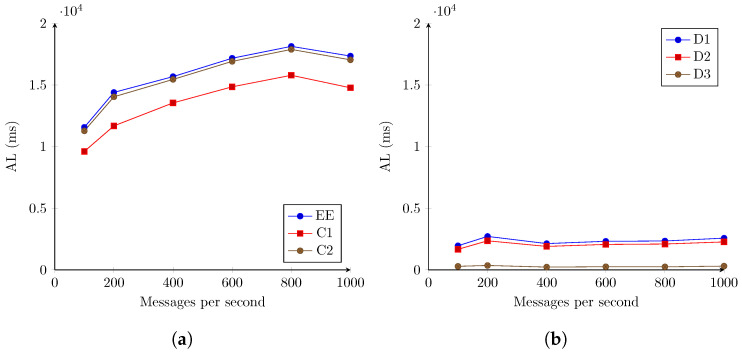
AL registered for the second stage evaluation. LoRaWAN for 100 simulated devices. (**a**) Registered latencies for monitored components and end to end. (**b**) Differences in recorded latencies between components.

Figure 7 repeats the previous experiment but configuring the IoTAgent-LoRaWAN component with 100 simulated devices. While writing the messages, they were randomly assigned to a different device.

This modification did not result in any significant gain, with previously noted patterns repeated. The initial interaction (up to the C1 point) accounts for between 9606.834 ms and 14,765.746 ms of latency. All remaining components, which include most processing and interactions, account for only between 1955.563 ms and 2714.426 ms of latency until each aggregation is available for consumption. Of particular note is the fact that, as seen in Figure 7b, the differences in measured latencies are stable for all message rates, indicating that the observed growth of end-to-end latency is primarily the result of the unstable behavior between the IoTAgent-LoRaWAN and the OCB.

The reduction in average latency for 1000 messages per second is also confirmed, but reduced from 1356.975 ms to 785.422 ms. This inversion, for both scenarios, is interpreted as a result of the size used for batching by default, being 1000 messages for both Cygnus and Kafka Connect components, decreasing the tail latencies. This observation is supported by the error rates in the following subsection (Section 4.2.2), for the same scenario. The percentage of messages with total latency superior to 25 s is cut from 17.30% to 6.60%, while latency for shorter time periods remains unchanged.

**Figure 8:**
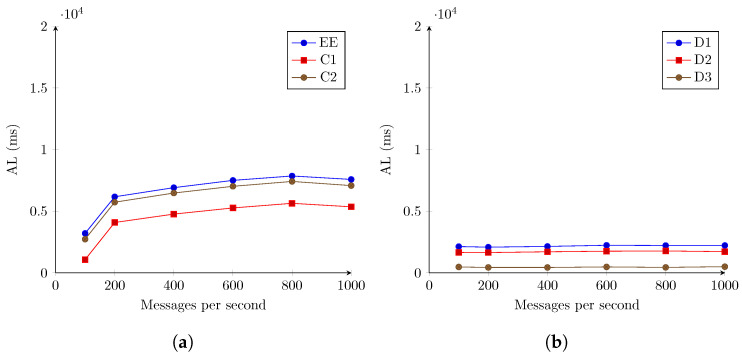
AL registered for the second stage evaluation. LoRaWAN for 100 simulated devices, and three instances of IoTAgent-LoRaWAN. (**a**) Registered latencies for monitored components and end to end. (**b**) Differences in recorded latencies between components.

Considering that previous research suggests that the Orion Context Broker should be capable of handling higher messages rates [45,46], the study of latency reduction was focused on the IoTAgent-LoRaWAN component. A third scenario was considered, where three instances of the IoTAgent-LoRaWAN component were used, each instance handling a third of the message volume, and communicating independently with the Orion Context Broker. One hundred simulated devices were once again considered. The results are shown in Figure 8.

This scenario, while similar to the previous results, presents a significant reduction in latencies for the C1 measurement point, and, therefore, for the total latency in the system. However, the IoTAgent-LoRaWAN instances continue to be the main performance bottleneck point.

Latency at the C1 point is now between 1074.035738 ms and 7577.968 ms, a decrease in the order of 8.945 and 2.756 times, respectively, in comparison to the scenario in Figure 7. The remaining components account for between 2083.829903 ms and 2235.426207 ms of latency. The IoTAgent-LoRaWAN instances continue to be the main performance bottleneck point.

While the decrease in latency for 1000 messages per second is still present, it is not as significant, at only 278.664 ms for the 785.422 ms in Figure 7.

#### 4.2.2. Error Rates

**Figure 9:**
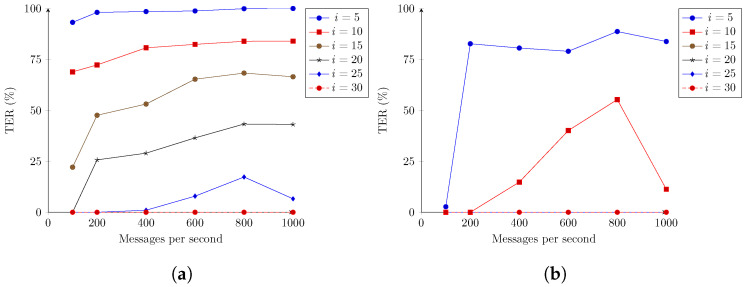
TER registered for the second stage—evaluation. (**a**) The 100 simulated devices and 1 IoTAgent-LoRaWAN, as in Figure 7. (**b**) The 100 simulated devices and 3 IoTAgent-LoRaWAN, as in Figure 8.

Figure 9 shows the error rates (TER) for the same set of experiments as Figure 7 and Figure 8, but now displaying error rates as defined in Equation (1) for different values of *i* seconds. As defined in Equation (1), the TER is considered to be the number of messages the system fails to deliver in *i* seconds, and therefore are considered lost. For all tests, there was no full message loss—a message not being delivered in any time period.

Comparing the results obtained for Figure 9a,b, the reductions in latencies, previously obtained by increasing the instances of IoTAgent-LoRaWAN from 1 to 3, resulted in predicable reductions in the error rates. In particular, there were no messages delivered after the time limit, for i=15, while previously this was only achieved for i=30. For 1000 messages per second, in Figure 9a, there is a clear reduction in tail latencies (messages with delivery latencies above 20 s). The same effect is also noted, in Figure 9b, for 10 s, with a more significant drop from 55.30% to 11.30%. As stated in the previous subsection, this drop is understood to be related to internal batching size used in the scenarios.

Considering the listed requirement that the system cannot fail to deliver more than 1% of messages, all scenarios could be considered to have kept the requirement, given a maximum limit of 30 s of delay, but failed this requirement considering a limit of 5 s. Even the best obtained case, the 100 messages per second mark for Figure 9b, had a TER of 2.7%.

## 5. Conclusions

The presented work proposes the architecture, and reference implementation, for a new system for collecting raw reports on metrics which are made available through a web application programming interface (API), and a corresponding user interface, displaying a heatmap. The system is built around components belonging to the FIWARE project, while considering existing integration with external data sources and processing systems. To the best knowledge of the authors, there is no previous solution that uses FIWARE components to gather metrics on the LPWAN deployments themselves that could be used to monitor network coverage and availability, which by themselves could be used to establish strategies to improve the network throughout its lifetime.

Validation was successfully carried out using real data collected from the city environment in Lisbon, Portugal, to obtain a visualization of the LoRaWAN network available in the city. The authors, however, believe that their proposed architectural approach is applicable to any urban environment, as it relates to how IoT devices and the LPWAN infrastructure perceive the channel characteristics in practice, over time. Further validation was carried out through the evaluation of processing latencies and error rates, using a simulated data set fed directly into the architecture. The evaluation process found that, while the objective of avoiding the loss of messages was successfully achieved, the system presents significant latency in processing the messages. Most of this delay is introduced by the IoTAgent-LoRaWAN component, used as reference implementation for the IoTMapperLpwanReceivers. Increasing the number of IoTAgent-LoRaWAN instances and splitting messages reception among them reduced latency between 8.945 and 2.756 times.

The authors found that, overall, the FIWARE components are adequate for building solutions with the given objectives, considering that most of them handled the messages efficiently. However, the limitations regarding latency, inserted by IoTAgent-LoRaWAN, represents a significant challenge to be overcome. Additional work is required, either by identifying and resolving internal bottlenecks that prevent it from successfully processing multiple messages concurrently or identifying appropriate alternatives. For example, as a solution for internal bottlenecks, mechanisms such as adding support for batching mechanisms already supported by Orion Context Broker may be added. The present architecture and implementation were also found to be valid as an initial answer to the listed requirements but may require additional validation and improvement, in particular, fine tuning batching sizes used in the different components, the performance of processing steps carried out by Kafka Streams, and considering multiple implementations for the IoTMapperLpwanReceivers.

The authors consider that there are three major drawbacks that affect the presented architecture for IoTMapper. Firstly, the chosen architecture is centered on the challenges surrounding active monitoring of LPWAN Networks. As such, this method can only be used to plan improvements to deployments in cities with existing networks; planning for completely new deployments must refer to other methods. Secondly, the system requires either regular survey work to update the aggregations or that devices used in deployed solutions are voluntarily provided. Lastly, the analysis to be carried out is limited to what metrics are made available for each network. For LPWAN deployments that depend on mobile network operators, for example, NB-IoT, such metrics may be severely limited without integration with each operator.

Throughout the paper, no consideration was made for completely replacing the FIWARE components, in particular, the Orion Context Broker that is the fundamental context management component of FIWARE. This is the result of one of the main design requirements: the usage of FIWARE components in order to validate their applicability to the problem area. To obtain a more comprehensive view of the performance of FIWARE, the authors suggest, as a future research possibility, comparison with other technological components.

## Figures and Tables

**Table 1:** Machines used for deployment and validation.

Legend	Description	CPU	Memory	Operating System
TFM-00	Deploys the *Input Layer*	4 vCPU	16 Gb	Ubuntu 22.04 LTS (5.15.0-40-generic)
TFM-01	Deploys the *Exit Layer*	4 vCPU	8 Gb	Ubuntu 22.04 LTS (5.15.0-40-generic)
TFM-02	Runs test loads	4 vCPU	8 Gb	Ubuntu 22.04 LTS (5.15.0-40-generic)

**Table 2:** Mappings for figure legends, defining measuring points for latency.

Legend	Name	Description
EE	End-To-End	Latency measured from message creation to being written as an aggregation in the Orion Context Broker (OCB)
C1	Component 1	Latency measured at the moment the IoTAgent-LoRaWAN finishes sending each message to the OCB
C2	Component 2	Latency measured at the moment each aggregation is written to Apache Kafka
D1	Difference 1	Difference in latencies measured in EE and C1. Latency for all components, but IoTAgent-LoRaWAN
D2	Difference 2	Difference in latencies measured in C1 and C2. Latency spent processing the message into an aggregation
D3	Difference 3	Difference in latencies measured in EE and C2. Latency spent inserting each aggregation into the OCB

## Data Availability

Not applicable.

## Appendix A

This appendix lists the data models defined for usage with FIWARE components, confirming NGSI-v2.

### Appendix A.1

Representation for generic and raw message entity.

MetricsReport: description: ’Description of a generic and raw message entity, containing a message coming from a device or other data source.’ properties: dateCreated: description: ’Entity creation timestamp. This will usually be allocated by the storage platform.’ format: date-time type: string x-ngsi: type: Property dateModified: description: ’Timestamp of the last modification of the entity. This will usually be allocated by the storage platform.’ format: date-time type: string x-ngsi: type: Property id: anyOf: - description: ’Property. Identifier format of any NGSI entity’ maxLength: 256 minLength: 1 pattern: ’^[\w\-\.\{\}\$\+\*\[\]`|~^@!,:\\]+\$’ type: string - description: ’Property. Identifier format of any NGSI entity’ format: uri type: string description: ’Unique identifier of the entity’ x-ngsi: type: Property name: description: ’The name of this item.’ type: string x-ngsi: type: Property MetricsReport: description: ’The raw value of the report, represented as a string. Must be URL safe.’ type: string x-ngsi: model: https://schema.org/Text type: Property type:

### Appendix A.2

Representation for an aggregation of metrics from some LPWAN data source.

MetricsAggregation: description: ’Description of a generic aggregation of measures/metrics’ properties: dateCreated: description: ’Entity creation timestamp. This will usually be allocated by the storage platform.’ format: date-time type: string x-ngsi: type: Property dateModified: description: ’Timestamp of the last modification of the entity. This will usually be allocated by the storage platform.’ format: date-time type: string x-ngsi: type: Property id: description: ’Property. Identifier format of any NGSI entity’ maxLength: 256 minLength: 1 pattern: ’^[\w\-\.\{\}\$\+\*\[\]`|~^@!,:\\]+$’ type: string x-ngsi: type: Property name: description: ’The name of this item.’ type: string x-ngsi: type: Property type: description: ’NGSI Entity type. It has to be MetricsAggregation’ enum: - MetricsAggregation type: string x-ngsi: type: Property dateLastGwUpdate: description: ’Timestamp of the last action that led to the rest of the this aggregation’ format: date-time type: string x-ngsi: type: Property dateLastUpdate: description: ’Timestamp of the last measure aggregated’ format: date-time type: string x-ngsi: type: Property gwId: description: ’Unique identifier of the Gateway’ maxLength: 256 minLength: 1 pattern: ’^[\w\-\.\{\}\$\+\*\[\]`|~^@!,:\\]+$’ type: string x-ngsi: type: Property mapTile: description: ’Unique identifier of the geographic area’ maxLength: 128 minLength: 1 pattern: ’^[\w\-\.\{\}\$\+\*\[\]`|~^@!,:\\]+$’ type: string x-ngsi: type: Property measures: description: ’List of aggregated measures’ type: array items: properties: name: type: string value: type: double type: object location: description: ’Geojson reference to the location covered by the item. It can be Polygon or MultiPolygon’ oneOf: - description: ’Geoproperty. Geojson reference to the item. Polygon’ properties: bbox: items: type: number minItems: 4 type: array coordinates: items: items: items: type: number minItems: 2 type: array minItems: 4 type: array type: array type: enum: - Polygon type: string required: - type - coordinates title: ’GeoJSON Polygon’ type: object - description: ’Geoproperty. Geojson reference to the item. MultiPolygon’ properties: bbox: items: type: number minItems: 4 type: array coordinates: items: items: items: items: type: number minItems: 2 type: array minItems: 4 type: array type: array type: array type: enum: - MultiPolygon type: string required: - type - coordinates title: ’GeoJSON MultiPolygon’ type: object x-ngsi: type: Geoproperty required: - id - type - gwId - mapTile - measures - location type: object version: 1.0.0
